# Remarks on the Cause and Treatment of Yellow Fever and the Bedford (Va.) Springs Alum and Iron Mass as a Remedial Agent

**Published:** 1878-10-20

**Authors:** W. A. Greene

**Affiliations:** Macon, Ga., Ex-President Georgia Medical Association; formerly chief Surg. of Artillery, 3rd Army Corps, A. N. Va.


### SOUTHERN MEDICAL RECORD.

VOL. VIII. ATLANTA, GA., OCT. 20, 1878. NUMBER 10.

EDITORS:

THOMAS S. POWELL, M. D. W. T. GOLDSMITH, M. D. R. C. WORD, M. D.

*R. C. WORD, M. D., Managing Editor.*

SUBSCRIPTION—$2.00 PER ANNUM, IN ADVANCE.

☞ All Communications and Letters on Business connected with THE RECORD, for 1877 and 1878, must be addressed to the Managing Editor.

### *ORIGINAL AND SELECTED.*

### REMARKS ON THE CAUSE AND TREATMENT OF YELLOW FEVER AND THE BEDFORD (VA.) SPRINGS ALUM AND IRON MASS AS A REMEDIAL AGENT.

By W. A. Greene, M.D., Macon, Ga., Ex-President Georgia Medical Association; formerly chief Surg. of Artillery, 3d Army Corps, A. N. Va.

The great pestilence of 1878, which "walketh in darkness and destroyeth at noon" may be stated in round numbers to have extended over five hundred miles in length, on the Air-Line, and seventy miles across at its widest part, sparing neither city, town, village or hamlet, clinging to the eastern side of the Mississippi river—it greatest divergence being at Chattanooga; Mobile, Charleston, Savannah and Jacksonville, and other places subject to its visits being free to this time. It seems to prefer moving Northward along the rich malarial pastures of the vast lowlands that stretch out from the turbid Father of waters.

In contemplating this wide-spread epidemic, and the terrible destruction of human life, our minds linger—to think of and admire the heroism and dauntless courage of those noble volunteers and martyrs, who, while others have fled before the approach of the pestilence, have remained by the noisome couch, have breathed the foul breath, and inoculated themselves with the deadly secretions of disease, to mitigate the sufferings or save the lives of their fellow beings—and wrest from the grasp of the poison some salutary instruction.

For these two long, weary months, they have watched by the gray dawn, through the noontide heats, and all the live-long night, at the bed side of the sick, to catch the first ray of hope, to seize upon the first favourable moment in which to offer kindly aid and gain an advantage over the deadly monster.

Such entire and unreserved immolation of self, such devotion to the calls of duty —are but the repetitions of the acts and examples of thousands of our brethren, who have thus, like *Curtius* leaped into the gulf, and thrown away their own lives for their fellows; and in every age, and in every nation, are so numerous that their biographies would fill another Alexandrian Library. Like the *Prometheus* of the Greek Poet, they seem to struggle against Fate herself, and to traverse with unbending resolution the evil current of those who preceeded them to the same destiny. It is ours to stand ready to aid them with all our resources of study and investigation—that they may put limits to the sway of this dread king of terrors.

It is now too late to discuss clean streets and alleys, or correct sewerage or any system of hygenic measures—but rather, by experimental science and study of the true pathology of the disease, *seek to cure it. This is our only hope.* Our oft scourged Southern cities have already exhausted every resource, to prevent *its appearance,* until it seems no human foresight, or expenditure of time, talent or money will ever avail anything. And I regret to say, the frightful mortality in the present epidemic proves how unsuccessful and uncertain are our therapeutical means. We have in a measure mastered other diseases of nearly equal fatality as pulmonary consumption, Typhoid fever, small-pox, now a disease of the past. Rheumatism an opprobrium, now yields almost as certainly to modern treatment as the intermittents and remittents of malarial regions. In the present epidemic of yellow fever the oldest traditional theories have been demolished, and it seems to me that we are in greater ignorance of essential and specific knowledge than ever before.

Yellow fever looms up before the American people as a national peril and calamity, the Leviathen of our prosperity and the destroyer of the lives of our people, and invokes at once the serious attention of the government. It invades alike the palaces of the rich, and the hovels of the poor; in country as well as city; the inland cities and towns, as well as the great marts of trade, the water courses and thoroughfares of commerce—sparing neither age nor sex; the fœtus in utero, the babe at the breast, the youth, and the man alike are its victims. Neither is it a respector of nationalities, the hitherto exempted negro is almost equally affected with his "white brother." And it is a noticeable fact in the present epidemic, that previous attacks have proven less effective against a recurrence than ever before, as is evidenced from the numerous attacks and deaths among the physicians, druggists and nurses, who so nobly volunteered their services to the afflicted cities. Finally—although the flower and skill of the profession, have, at the imminent peril of health and life labored most zealously, yet, no treatment has been able to reduce the average mortality below thirty-five per cent. of those attacked.

I do not propose, in this paper, to discuss the pathology of yellow fever, but give simply a brief synopsis of my opinion of its direct cause and treatment. I very much fear the space allotted in a medical journal will be insufficient to make myself understood, or convey the probable benefit I venture to hope for, from a publication of my theory.

Yellow fever is a specific malignant fever, usually of a continued type, and propagated by minute germs, and has appeared at elevations of 2,000, and even 4,000 feet above the level of the sea. No physician who has studied the investigations of *Yellow* and *Malarial* fever, made by men of ability—or themselves made the invstigations, but have been struck with the *identity* of the two diseases in material respects, and I hold that any general treatment adapted to one will be found valuable in the other.

The most recent investigations agree with all previous ones, that yellow fever prevails exclusively in malarial regions, and is produced by spores of fungi floatin the air, and in which often, especially in mild types, remissions and intermissions of the fever are observable. Dr. LeHardy, of Savannah, who has given the subject perhaps more thought than any recent investigator, and fully competent, states that he has observed this (the remissions and intermissions) often among the negroes in the last Savannah epidemic, and in a majority of those cases quinine controlled the febrile symptoms. On this important point we have the valuable testimony of Dr. Robert Lawson, inspector general of hospitals, who has seen yellow fever on the West coast of Africa and in the West Indies, as well as the United States, and given special attention to the features which characterize it, and serve to distinguish it from other kinds of fever. He says, "the train of urinary symptoms and other characteristic features of yellow fever were as fairly developed in the periodical as in the continued form of the disease; the definition of yellow fever which represents it as being always of the continued form, is altogether erroneous."

While the yellow fever plant may not be identical with the malarial plant, (neither having been sufficiently isolated and explained); yet, the conditions for their development are strikingly similar, even to a casual observer. Neither are contageous by personal contact. Malarial fevers of more or less severe type invariably follow as a consequence, when there has been slight frosts, few freezes, unusual quantities of rain, saturating the earth and filling the ponds and low places, resulting in stagnant water and much decayed vegetable matter. These are the conditions which propagate yellow fever, and in which it luxuriates and delights to dwell, leaving wretchedness and death in its wake.

For these reasons and others, gathered from a residence and practice of ne rly thirty years in an intensely malarial region, with close observation and patient investigation of the diseases peculiar thereto, and an experience with yellow fever sufficient for practical purposes, I am forced to the conclusion that *it is the combined influence of the spores of the yellow fever and malarial plants, acting together, and upon each other, under favorable conditions for their mutual development*, which produce and propagate yellow fever. It will be noticed that the identical surroundings or conditions are always required for the germination, growth and development of yellow fever; and in closely studying and observing the types of different epidemics, I have noticed that it is controlled by the predominating presence of the one or the other cause, being milder if the *malarial* is in excess, and severer if the *yellow fever* has supremacy, and will yield to a lower degree of temperature or spread and be susceptible of more rapid multiplication when it is higher, for the same reasons.

And again, the treatment that has been found successful in one epidemic failed perhaps in the next, in the same locality, which can be explained by the same theory. A notable instance being the epidemic of 1847 in New Orleans, in which the cases were controlled most satisfactorily by quinine; but the advocates of quinine treatment were doomed to a most unexpected disappointment in the severer epidemic of 1853.

In the former epidemic, in all probability the *malarial* cause was in excess, and accounting for the good results of quinine, upon which the physicians mainly depended. The epidemic of 1853, as all will remember, was unsually severe, and I have no doubt the *yellow fever* poison predominated to such a degree as to render the *malarial antidote* almost inert as a remedy. There seems to be a mutual intensification of these subtle poisons by their commingling in the atmosphere, and entering the animal system.

The striking similarity which yellow fever so often presents to the malignant or pernicious forms of malarial fever, has always perplexed etymologists in the in-the investigation of the former—and this theory may open up the way for a more satisfactory elucidation of the subject. In fact so difficult is the defferential diagnosis, that the first cases in all epidemics are diagnosed and treated for some form of malarial fever, even by *experts*—those who have had considerable experience with yellow fever. It is fresh in the minds of many of us, who were residing in the Southern portion of Georgia at the time, concerning the doubts as to the character of the fever which proved so fatal in Bainbridge, Georgia, a few years since. For several weeks it was pronounced and treated for the *haemorrhagic malarial* fever which had been prevailing in that section previously---until the rapid multiplication of cases and alarming fatality excited and aroused the entire State, and consulting physicians were summoned from the yellow fever cities, and pronounced it genuine yellow fever. There are those, even to this day, and intelligent physicians, too, who are not satisfied with this diagnosis. Bainbridge is a small inland town, with some 1500 inhabitants where yellow fever had never before appeared---neither was it at the time prevailing at other points in the vicinity, so far as I remember, nor did it extend beyond the town. There was also considerable speculation concerning the fever when it first appeared in Memphis in the epidemic before this one. In neither of these instances did the fever yield as promptly to the *first* frosts as has been its custom on the *coast* heretofore. We can here again explain these irregularities on my theory of the two poisons operating together, and the malarial predominating---for we know the latter does not yield decidedly to low temperatures—-but is decidedly mitigated.

In the fever called "Typho-malarial" we have another instance of two separate and distinct poisons operating at the same time, in the same patient---the typhoid and malarial fungi. We have a knoweldge of the typhoid fever fungus, which is distinct from the malarial plant. According to the predominance of the one or the other of these causes, will the fever assume the typhoid or the malarial characters in excess, and to be treated accordingly.

The haemorrhagic malarial fever, so similar to yellow fever, requiring nearly the same treatment, I believe to be caus-by the spores of a plant resembling the yellow fever plant, which, combining with the malarial plant, intensify each other, and poison the blood of those coming within its influence, producing this characteristic fever, which, of recent years, has prevailed so extensively and fatally in the malarial regions of the South.

Since this fever made its appearance in Georgia during the years 1868 and 1869, I have carefully and patiently watched and studied it, with a view of ascertaining its true cause, pathology and treatment. I have given the profession the benefit (if any) of what little I have discovered in previous papers, contributed to the "Richmond and Louisville Medical Journal," in 1870, and more recently in "The St. Louis Medical and Surgical Journal" (June number, 1878), and the "Cincinnatti Medical News," October, 1878. In those papers I strongly advocated the use of the "Alum and Iron Mass" as a chief remedy, sustained by giving a few cases. It was in pursuing those investigations I arrived at the theory of the cause of yellow fever as expressed in this paper, and from the strong resemblance of the two diseases, based my theory of the treatment ofyellow fever. I regret my limited opportunities for more thoroughly testing my views of this treatment of yellow fever with the iron and alum mass, before appearing before the profession in an arti cle so strongly recommending it; and would not do so, but for the reason that the disease is prevailing so alarmingly and fatally in our Western cities, and if there is any good in it, the physicians there could use it, test it as thoroughly and perfectly as I could, and more so; and if my sanguine expectations should be realized, even a *tithe* of them, I will feel that I have done some good, though not present to share with them the dangers and hardships of administering directly to the afflicted ones. I can say, truthfully; I never had more confidence in a remedy, and have good reasons for it, from my experience with it, and success in treating haemorrhagic malarial fever, and the few cases of yellow fever that came under my notice and attention in 1876, from Savannah. I earnestly insist that the physicians who now have ample opportunities for testing the remedy, will do so at once, and communicate directly with me for any further information on the subject. But for my peculiar surroundings, I would at once go to some portion of the yellow fever district, and use this theory of treatment, and make further investigations of the causes.

I invite a careful examination of the analysis of the mass as procured from the waters of the Bedford, (Va.,) springs, which, taken in consideration with my theory of the cause of yellow fever, and its similarity to the form of malarial fever I have considered, the adaptability of the treatment at once becomes apparent. The usual preliminary treatment of putting the patient to bed, covering him up, closing the apartment, foot baths, with strong mustard and pepper, friction of limbs, elder-leaf and other teas to produce perspiration, assisted by hot bricks, bottles of hot water, and boiled corn, about patient's body and extremities, or mustard plasters *ad infinitum*, may sometimes be proper in a guarded manner. But this hot, fiery, pungent treatment, should be carefully administered, as every case may not require this stereotyped proceeding.

In all specific diseases there is some specific remedy on which we build our main hope of reliance as the alkaloids of Cinchona bark in intermittents and remittents, mercury in Syphilis, Iod Potass and Salacylic Acid in Rheumatism, etc. In yellow fever, I should build my hope of cure chiefly on the Iron and Alum Mass of the Bedford (Va.,) Springs, as it is condensed from the water by pure and simple evaporation. It is now well-known to all physicians and needs no special mention. The medical properties of the several constituents as shown by the analysis of eminent chemists will readily suggest their effects on the system. It is an alterative tonic, steadfast and gentle diuretic and febrifuge, mild aperient, acting alike on the secretary and excretory organs—and when administered in proper doses producing as satisfactory, "billious discharges" from the bowels, as are obtained from calomel. I regret the lack of space to speak more particularly of the analysis and consider in detail the medical properties and therapeutical effects of each constituent, I will however refer to some of the most prominent ones. We notice first, the unusual quantity of iron existing in the mass chiefly as ferric sulphate, the most useful and valuable form for medicinal purposes. Also the sulphate of aluminum. The sulphate of magnesia and sulphate of manganese are very valuable properties, the latter just now attracting much interest since Dr. Goolden, of the London *Lancet*, described its peculiar and specific virtues as a remedy substituting calomel, for its direct action on the liver, and free from many objections of this powerful drug, in debilitating affections. When combined with sulphate of magnesia (epsom salts) as in these waters, it requires very minute quantities to obtain its specific action. It is principally to these ingredients we are indebted for the action of this "mass" on the hepatic secretions. Then again we find a large compliment of free sulphuric acid. In the treatment of hæmorrhagic malarial fever, I have for a long time considered the *mineral tonics* absolutely required in its treatment, and I find Professor Jones in the "New Orleans Medical and Surgical Journal" recently commending them in the treatment of yellow fever, also, which is increased and most valuable testimony in support of my theory of treatment of the two diseases, and also, of the further position that a similar treatment is adapted to each. The mineral tonics abound in this combination of the Alum and Iron Mass, containing ten parts of iron to every hundred of the mass. The other constituents which assist materially in promoting its good effects in various diseases, I have not time to consider in detail.

If these views briefly and imperfectly advanced as to the abnormal conditions existing in yellow fever be correct, the indications of treatment would be mineral tonics to produce contractility of the capillary vessels, and effect needful changes in the blood which has been prevented by the poisons, to stimulate the functions of the kidneys, bowels and skin to increased activity, and, to interrupt any paroxysmal disposition or tendency of the fever. I have not had trouble with suppression of urine, since using this remedy, in the treatment of these diseases, unless the case was too far advanced before treatment.

My mode of administering the Alum and Iron Mass is to begin as early in the attack as possible, giving the largest possible doses the patient will tolerate, saturating the blood, and bringing the system speedily under its influence, and afterwards continue it in such doses as may be necessary to preserve its effects; preceeded by a warm pedeluvium, or, in bad cases, a semicupium. In the meantime make use of such hygienic treatment and regulations as the general indications may require. These, of course, are not regular in all cases, but are governed by all the various circumstances and conditions that regulate us in the treatment of any other disease. If the medicine is properly administered and the patient well nursed under ordinary circumstances, very soon the pain in head, back and limbs will begin to subside, and the nausea be relieved—the urine rapidly improves, losing its thick, albuminous charactei, gradually becoming clear and limped, and the paroxysms are rendered milder, less frequent and of shorter duration. Of course this is the result in favourable cases of yellow fever, and in Hæmorrhagic malarial fever may be looked for oftener than otherwise.

Of the *modus operandi* of the "Alum Iron and Mass" and the mineral waters of the Bedford (Va.) Springs, I confess knowing but little if anything; and yet, perchance this may be as much as any of us know in relation to the *modus operandi* of many other articles in the relief of diseases for which they are so confidently administered.

The author of the above idea further says, that each and every organ and tissue in the animal economy is possessed of a *vires vitae*, which *vires vitae* is peculiar to and inherent in such organ or tissue. That this *vires vitae* is susceptible of being acted upon, stimulated or depressed by appropriate agents, follows as a necessary consequence. Add to this, the well known established principle in therapeutics, that each remedy in the great arcana has some inherent property or quality that directs its action to one organ or tissue in preference to another; in other words, it is possessed of an elective affinity or franchise which directs, controls or modifies its action; and we derive our knowledge of this affinity from accident, from experience, or after a chemical analysis. We venture to use it when there appears to be a natural adaptation to the pathological condition of the organ or tissue. From these aphorisms, pathological and therapeutical, we may be able to deduce the *modus operandi* of the Alum and Iron Mass in yellow fever and hæmorrhagic malarial fever.

If the foregoing propositions and suggestions, hastily and very imperfectly considered, contain any considerable amount of truth, it behooves us, as the guardians of suffering humanity, to propose better ones, or adopt them at least for a trial.

I am not able to furnish an egotistical array of cases of yellow fever, but only make a simple statement of a few *facts* in regard to the effects of this remedy in the disease under consideration. I hope no one will be so unkind as to denounce the remedy on account of the humility of its origin. I commenced using it in cases of the diseases mentioned and found I was much more successful with it than with any former course pursued by me. I have instituted comparative tests with it and other remedies recommended by our best authors, and yet prefer the one under consideration. I am aware, and it is proper, that facts, ascertained by experiments sufficiently clear, and repeated sufficiently often, and observed by a sufficient number of witnesses, must be taken as the basis of all the sciences--and of ours more than all the rest.

Every theory appeals at last to facts and experience: there is no alternative, and must consent to be judged by the observed results of the application of the principles or hypothesis suggested. The powerful force and reliability, as well as confidence of our ancient brethren in the results of experience and experiment as being conclusive of the value of any remedy—we find in the sayings of Brousais, who was the most ingenious and obstinate of dogmatists. He said in his forcible strictures upon the numerical plan of Louis that, "If it can be proved upon experience that tartar emetic will cure a *gastro enterite*, I will administer it; if arsenic will do good, I will prescribe that." This, then we may recognize, as the primary rule, the elementary principle of our science. From the champion of every theory or school we should be willing to learn what he is eager and able to teach—and to select from each his best weapons and make them useful in this interminable contest, and as in the phrase of Bacon, "all error is founded in some truth" we can find instruction everywhere.

In concluding this paper I desire to say further concerning the use of the Alum and Iron Mass—that it is a very valuable remedy in many forms of cutaneous affections, more especially of the scaly variety. I have seen cases of Psoriasis inveterata which had resisted the long continued use of arsenic, iodine and other remedies, yield readily to its influence, especially in patients of dissipated habits, complicated with enlarged liver. It is also a useful remedy in eczema and syphilitic squamæ; open ulcers which have long resisted other treatment I have seen heal rapidly by its local application—at same time taking it internally.

In the various forms of dyspepsia, I have observed the most gratifying results, speedily relieving the severe headache caused by deranged digestion and inactive liver.

The following extract from a private letter written me by Bishop Geo. F. Pierce, of Georgia, is valuable testimony of the efficiency of alum and iron mass, for the troubles he mentions and from which many of our public men suffer in the South, who perform large amounts of brain work, as does this highly favored, eloquent, and universally beloved Bishop.

He says—"I knew one extreme case of dyspepsia cured by the iron and alum mass. It cures ordinary sick headache, will prevent it if taken in time. I found it in my own case when greatly run down in strength, an excellent tonic restoring appetite and vigor. I have great faith in it, in all cases for which it is recommended." If any reliance can be placed on what I have written concerning Alum and Iron Mass, it is unquestionably a valuable medicine, possessing active curative powers, and having a wide range of action. The supply being inexhaustible, easily procured, and cheap—is more likely to be pure and of uniform strength. It is peculiarly adapted to hospital and dispensary practice, and should attract more attention from the profession in this country than has hitherto been given to it.

---

